# Molecular Characterization of *TSC1* and *TSC2* Variants in a Greek Cohort of Tuberous Sclerosis Complex Patients

**DOI:** 10.1155/humu/4508699

**Published:** 2026-08-10

**Authors:** Socratis N. Avgeris, Florentia Fostira, Paraskevi Apostolou, Angeliki Delimitsou, Stefanos Smyrniotis, Drakoulis Yannoukakos, Dimitrios J. Stravopodis, Popi Syntichaki, Gerassimos E. Voutsinas

**Affiliations:** ^1^ Laboratory of Molecular Carcinogenesis and Rare Disease Genetics, Institute of Biosciences and Applications, National Centre for Scientific Research (NCSR) “Demokritos”, Athens, Greece; ^2^ Human Molecular Genetics Laboratory, INRASTES, National Centre for Scientific Research (NCSR) “Demokritos”, Athens, Greece; ^3^ Section of Cell Biology and Biophysics, Department of Biology, National and Kapodistrian University of Athens (NKUA), Athens, Greece; ^4^ Biomedical Research Foundation of the Academy of Athens (BRFAA), Athens, Greece

## Abstract

Tuberous sclerosis complex (TSC) is an autosomal dominant multisystem genetic disorder caused by pathogenic variants in the *TSC1* or *TSC2* genes, resulting in dysregulation of the mTOR signaling pathway and subsequent hamartoma formation. Although the genetic basis of TSC is well established, population‐specific data on *TSC1* and *TSC2* variants are still emerging. The aim of this study was to characterize the variant spectrum of the *TSC1* and *TSC2* genes in a cohort of 34 unrelated probands from Greece, 26 of whom had a definite TSC diagnosis, whereas eight had a possible TSC diagnosis. Targeted next‐generation sequencing (NGS) was performed to analyze all coding exons and flanking exon–intron boundaries of *TSC1* and *TSC2*. Identified variants were subsequently validated by Sanger sequencing. Pathogenic or likely pathogenic variants were identified in 22 of 34 probands, corresponding to an overall diagnostic yield of 65% using the testing strategy applied in this study. Of these variants, 32% (7/22) occurred in *TSC1* and 68% (15/22) in *TSC2*; seven variants (7/22; 32%) were previously unreported. The molecular detection rate was 77% (20/26) for patients meeting the criteria for definite clinical TSC diagnosis and 25% (2/8) for those with a possible TSC diagnosis. Exploratory genotype–phenotype analysis revealed a trend toward a more severe clinical presentation among patients harboring *TSC2* variants. These findings expand the known molecular landscape of TSC and support the clinical utility of genetic testing for diagnosis, genetic counseling, and patient management.

## 1. Introduction

Tuberous sclerosis complex (TSC) is a rare genetic disorder inherited in an autosomal dominant manner. It is characterized by considerable clinical heterogeneity and the development of multiple benign hamartomas in various organ systems, including the brain, skin, heart, liver, kidneys, lungs, and eyes [1]. However, given the broad spectrum of clinical features, no single feature is pathognomonic for the condition. At the molecular level, TSC is caused by the presence of pathogenic variants in either the *TSC1* (located at 9q34) or the *TSC2* (located at 16p13.3) gene, which encode the proteins hamartin and tuberin, respectively [2, 3]. These proteins associate with TBC1D7 to form a multimeric complex that functions as a critical negative regulator of the mTORC1 pathway, an evolutionarily conserved master kinase complex that regulates a multitude of cellular processes, such as translation, autophagy, ribosome and macromolecule biogenesis, and neoangiogenesis [4, 5]. Defective function of either the *TSC1* or the *TSC2* gene leads to inadequate regulation of cell growth and proliferation, manifesting as the TSC phenotype. The ubiquitous expression of hamartin, tuberin, and mTOR across numerous tissues and organs explains the multisystem involvement characteristic of TSC [6]. Despite considerable advances in elucidating the molecular characterization of TSC, comprehensive data regarding the spectrum of *TSC1* and *TSC2* variants in southeastern European populations remain limited, particularly for Greece. Furthermore, although genotype–phenotype correlations have been reported, their clinical significance is subject to continuous refinement. Therefore, the present study is aimed at characterizing the molecular spectrum of *TSC1* and *TSC2* variants in a Greek cohort of patients with TSC using next‐generation sequencing (NGS) to identify previously unreported variants and explore potential genotype–phenotype correlations.

## 2. Materials and Methods

### 2.1. Patients and Ethics Statement

The study protocol and informed consent forms were approved by the Bioethics Committee of NCSR “Demokritos” (Approval No.: 240/2016‐922). Patients were referred to the Laboratory of Molecular Carcinogenesis and Rare Disease Genetics for genetic screening between 2017 and 2018. Written informed consent was obtained from all probands or their legal guardians/parents; following analysis, families were informed in detail regarding the clinical significance of the genetic test results. Finally, the study was performed in full compliance with the principles of the Declaration of Helsinki of 1975, as revised in 1983.

### 2.2. Variant Detection and Confirmation

Genomic DNA was extracted from peripheral blood using a standardized high‐salt/chloroform extraction protocol [7]. The concentration and purity of the isolated genomic DNA were quantified using a NanoDrop 1000 spectrophotometer (Thermo Fisher Scientific, Waltham, Massachusetts, United States). The integrity of genomic DNA was simultaneously evaluated via agarose gel electrophoresis.

Targeted NGS was performed on the *TSC1* and *TSC2* genes using the Illumina MiSeq System (San Diego, California, United States). The study utilized a 94‐cancer gene panel (TruSight Cancer Panel, Illumina, San Diego, California, United States), which included oligonucleotide probes designed to cover all exonic regions and ±20 base pairs (bp) of the flanking intronic sequences for each targeted gene.

Amplified libraries were evaluated qualitatively and quantitatively using Fragment Analyzer (Advanced Analytical Technologies, Heidelberg, Germany). Indexed libraries were sequenced on MiSeq using the Reagent kit V2 performing 150 base paired‐end reads, whereas FASTQ, BAM, and VCF files were generated through Illumina MiSeq Reporter; annotation was performed against the human reference genome GRCh37/hg19 using Variant Studio V3.0 (Illumina). The minimum base and amplicon coverage were 50× and 100×, respectively, whereas the mean read depth was 182× [8].

The targeted NGS workflow implemented in our laboratory has a validated analytical limit of detection (LoD) of approximately 10% variant allele frequency (VAF). Accordingly, variants with a VAF of ≥ 10% were considered for downstream interpretation. Although the assay was developed and clinically validated primarily for constitutional germline testing, its analytical sensitivity enables the identification of variants present at allele fractions of approximately 10% or greater; therefore, low‐level mosaic variants below this threshold cannot be reliably excluded.

Transcripts NM_000368.4 and NM_000548.3 were used as reference sequences for the *TSC1* and *TSC2* genes, respectively. All sequence variants were described according to the recommendations of the Human Genome Variation Society (HGVS) nomenclature guidelines [9].

All NGS‐identified variants were confirmed by Sanger sequencing performed on an ABI 3130 XL Genetic Analyzer (Applied Biosystems, Foster City, United States) in the index cases and their available parents. Sequencing reactions were performed using the v3.1 BigDye Terminator Cycle Sequencing kit (Applied Biosystems, Foster City, California). All primer sequences and PCR conditions used are shown in Tables S1 and S2 (see supporting Materials and Methods).

### 2.3. In Silico Analysis

Identified variants were cross‐referenced against the Leiden Open Variation Database (LOVD) and ClinVar to evaluate established clinical significance. Variants of uncertain significance (VUS) were initially screened using the VarSome human genomic variant search engine and the Cancer Predisposition Gene Variant Database (CanVar‐UK v2.2) to assess potential pathogenicity. The functional impact of amino acid substitutions was predicted using PolyPhen‐2 and SIFT; insertions and deletions were evaluated using MutationTaster 2025. The potential impact of variants on pre‐mRNA splicing was evaluated using splice‐site prediction tools integrated within VarSome, specifically MaxEntScan and dbscSNV. To provide structural context for these predictions, hierarchical protein modeling was performed using the I‐TASSER server. Finally, population allele frequencies were determined using the Genome Aggregation Database (gnomAD). All variants were interpreted according to the American College of Medical Genetics and Genomics and the Association for Molecular Pathology (ACMG/AMP) guidelines [10].

### 2.4. Statistical Analysis

Statistical differences in proportions of genotype–phenotype correlations between patient groups were assessed using Fisher′s exact test due to the small sample sizes. The threshold for statistical significance was set at *p* < 0.05.

## 3. Results and Discussion

### 3.1. Patients′ Characteristics

Thirty‐five unrelated probands from Greece underwent germline genetic testing of the *TSC1* and *TSC2* genes. None of the probands included in the present study were part of our previously reported Greek TSC cohort [11]. One case was excluded because it failed to satisfy the revised criteria for a clinical diagnosis of TSC. The final cohort comprised 16 females (47%) and 18 males (53%).

The age at clinical diagnosis showed variability across the cohort: 38% (13/34) were diagnosed during adulthood, 32% (11/34) during childhood, 24% (8/34) during infancy, and 6% (2/34) during adolescence. Based on the revised diagnostic criteria for TSC [12, 13], 26 probands (76%) received a definite clinical diagnosis, whereas the remaining eight individuals (24%) presented with findings consistent with a possible TSC diagnosis.

Clinical characteristics were comprehensively collected from patient medical records and family reports. Data were derived from extensive evaluations performed by various medical specialties, including diagnostic imaging (computed tomography, magnetic resonance imaging, ultrasonography, and echocardiography), electrophysiological assessment (electroencephalography), and clinical examinations (ophthalmoscopy).

The clinical characteristics of TSC patients are presented in Table S3. It should be noted that because several clinical manifestations exhibit age‐dependent penetrance and expression, the phenotype of younger patients or infants may potentially evolve and change as they age.

### 3.2. Characterization of Variant Pathogenicity

In the present study, genetic testing for *TSC1* and *TSC2* genes was carried out on 34 probands; the presence of pathogenic variants was investigated in DNA from both parents (except for Families 24 and 26, because of one deceased parent in each case) and, in some familial cases, from other family members as well (7, 11, and 12). Paternity was not interrogated, whereas mosaicism was not excluded for the parents of the apparent de novo cases. In total, 22 pathogenic/likely pathogenic variants were identified (Table 1). Of these, seven (32%) represent novel variants that have not been previously reported in the literature. Each was classified in accordance with the ACMG/AMP guidelines [10].

**Table 1:** Genetic characteristics of TSC probands from Greece in the present study.

P/ID	Sex/age ^∗^	Gene	Diagnosis	Variant	Codon change	Inheritance	ACMG criteria	LOVD ID	ClinVar ID	Novel	Classification
1	F/1	—	Definite	—	—	—	—	—	—	—	NMI
2	F/2	*TSC1*	Definite	c.1987G>T	p.Glu663Stop	De novo	PVS1, PS2, PP3	—	280309	—	Pathogenic
3	M/22	*TSC2*	Definite	c.42_68del26	p.Lys14Asnfs ^∗^12	Familial	PVS1, PP1, PP3	—	—	+	Pathogenic
4	M/44	*TSC2*	Definite	c.4493+1G>A	—	De novo	PVS1, PS2, PP3	TSC2_002671	373118	—	Pathogenic
5	F/38	*—*	Definite	—	—	—	—	—	—	—	NMI
7	M/10	*TSC1*	Definite	c.2302C>T	p.Arg768Cys	Familial	PM1, PM2, PP1, PP3	—	—	+	Likely pathogenic
8	F/35	*TSC1*	Definite	c.2101delC	p.Gln701Serfs ^∗^23	De novo	PVS1, PS2, PP3	TSC1_000303	48894	—	Pathogenic
9	M/1	*—*	Possible	—	—	—	—	—	—	—	NMI
10	M/10	*—*	Possible	—	—	—	—	—	—	—	NMI
11	F/35	*TSC1*	Definite	c.147C>G	p.Tyr49Stop	Familial	PVS1, PP1 moderate, PP4, PP3	—	1363358	—	Pathogenic
12	M/15	*TSC2*	Definite	c.982G>C	p.Ala328Pro	Familial	PVS1, PM1, PP1, PP3	TSC2_000809	—	—	Pathogenic
13	M/13	*TSC2*	Definite	c.1600delG	p.Val534Stop	De novo	PVS1, PS2, PP3	TSC2_003101	—	—	Pathogenic
14	F/38	*—*	Possible	—	—	—	—	—	—	—	NMI
15	M/6	*TSC1*	Definite	c.2041+1G>C	—	De novo	PVS1, PS2, PP3	—	64792	—	Pathogenic
16	M/30	*TSC2*	Definite	c.2161delG	p.Val721Cysfs ^∗^50	De novo	PVS1, PM6, PP3	—	—	+	Pathogenic
17	F/11	*TSC2*	Definite	c.4078G>T	p.Glu1360Stop	De novo	PVS1, PS2, PP3	TSC2_002258	65003	—	Pathogenic
18	M/10	*TSC2*	Definite	c.2212dupT	p.Cys738Leufs ^∗^24	De novo	PVS1, PM6, PP3	—	—	+	Pathogenic
19	M/5 m	*TSC2*	Definite	c.5238_5255del18	p.His1746_Arg1751del	De novo	PVS1, PS2, PM2, PP3	TSC2_000149	12402	—	Pathogenic
20	F/1	*TSC2*	Possible	c.975+2T>C	—	De novo	PVS1, PS2, PM1, PP3	—	1074350	—	Pathogenic
21	F/6	*TSC2*	Definite	c.1832G>A	p.Arg611Gln	De novo	PVS1, PS2, PP3	TSC2_000105	12397	—	Pathogenic
22	M/8	*—*	Definite	—	—	—	—	—	—	—	NMI
23	M/22	*—*	Definite	—	—	—	—	—	—	—	NMI
24	M/11	*TSC2*	Definite	c.3083_3091del	p.Asp1028_Met1030del	Familial	PM2, PM4, PP1, PP3	—	—	+	Likely pathogenic
25	F/43	*—*	Possible	—	—	—	—	—	—	—	NMI
26	F/20	*TSC2*	Definite	c.4762C>T	p.Gln1588Stop	Familial	PVS1, PM1, PP1, PP3	TSC2_000866	50097	—	Pathogenic
27	M/40	*—*	Definite	—	—	—	—	—	—	—	NMI
28	M/8 m	*TSC1*	Possible	c.663+1G>T	—	Familial	PVS1, PS1, PP1, PP3	—	—	+	Pathogenic
29	M/3	*—*	Possible	—	—	—	—	—	—	—	NMI
30	F/40	*—*	Definite	—	—	—	—	—	—	—	NMI
31	F/20	*—*	Possible	—	—	—	—	—	—	—	NMI
32	F/9	*TSC2*	Definite	c.888_889delGT	p.Phe298Cysfs ^∗^39	De novo	PVS1, PS2, PM1, PP3	TSC2_002485	—	—	Pathogenic
33	F/5	*TSC1*	Definite	c.1052_1053dupTG	p.Val352Trpfs ^∗^5	Familial	PVS1, PP1, PP3	—	—	+	Pathogenic
34	M/1	*TSC2*	Definite	c.1946+1G>T	—	De novo	PVS1, PS2, PP3	—	546435	—	Pathogenic
35	F/6 m	*TSC2*	Definite	c.4846C>T	p.Gln1616Stop	De novo	PVS1, PS2, PM1, PP3	TSC2_000788	49326	—	Pathogenic

*Note:* Asterisk “ ^∗^” denotes age of diagnosis in years or months; +/—: found/not found.

Abbreviations: NMI, no mutation identified; P/ID (Proband ID), each number corresponds to the specific proband; PM1, located in a mutational hot spot and/or critical and well‐established functional domain (moderate evidence); PM2, absent from controls or at extremely low frequency if recessive (moderate evidence); PM6, assumed de novo, but without confirmation of paternity and maternity; PP1, cosegregation with disease in multiple affected family members in a gene definitively known to cause the disease (supporting evidence); PP3, multiple lines of computational evidence support a deleterious effect on the gene or gene product (supporting evidence); PP4, patient′s phenotype or family history is highly specific for a disease with a single genetic etiology (supporting evidence); PS1, same amino acid change as a previously established pathogenic variant regardless of nucleotide change (strong evidence); PS2, de novo in a patient with the disease and no family history (strong evidence); PVS1, null variant (very strong evidence of pathogenicity).

The molecular diagnostic yield varied based on clinical certainty. Among the patients with a definite TSC diagnosis, 23% (6/26) had a pathogenic *TSC1* variant and 54% (14/26) had a pathogenic *TSC2* variant (*p* = 0.0256; calculated excluding patients with no pathogenic variant identified (NPVI)). In contrast, among the patients with a possible TSC diagnosis, 12% (1/8) had a confirmed *TSC1* pathogenic variant, and an equivalent percentage had a confirmed *TSC2* pathogenic variant.

Compared with familial cases (36%, 8/22), the majority of the identified variants were de novo (64%, 14/22). The frequency of de novo variants observed in the current cohort is similar to that reported in our previous study [11], which was 69%. Among the de novo cases, three (21%) occurred in *TSC1* and 11 (79%) in *TSC2* (*p* = 0.0070), whereas among the familial cases, four (50%) occurred in *TSC1* and four (50%) in *TSC2*.

**Figure 1:**
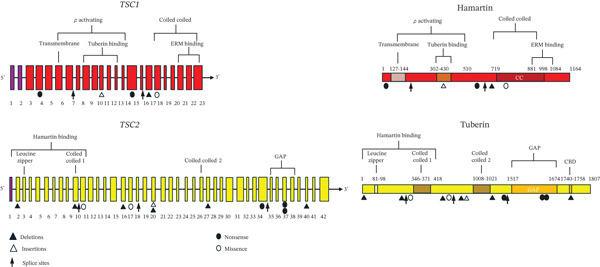
Distribution of the *TSC1* and *TSC2* variants identified in this study. Variants are categorized by type as indicated by the symbol legend: deletions, insertions, splice‐site, nonsense, and missense variants. Protein domains for hamartin (*TSC1*) and tuberin (*TSC2*) are indicated above the respective gene structures, whereas exon numbers are displayed below. Abbreviations: CBD, calmodulin‐binding domain; GAP, GTPase‐activating protein; ERM, ezrin‐radixin‐moesin.

**Figure 2:**
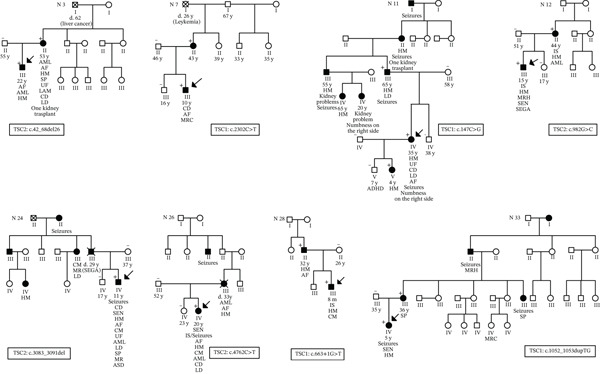
Pedigrees of the eight families studied. Arrows indicate the index patients (probands); symbols (+) and (–) denote the presence or absence of the identified variant, respectively. Clinical information for relatives was gathered through patient/relative interviews; formal medical records were only available for the probands. The identified pathogenic *TSC1* or *TSC2* variant for each family is indicated below the corresponding pedigree. Detailed definitions of clinical abbreviations are provided in the table of clinical characteristics (Table S3).

Regarding the variant spectrum, among the 22 identified pathogenic or likely pathogenic variants, seven were localized to *TSC1* (32%) and 15 to *TSC2* (68%), displaying a significantly higher frequency of pathogenic or likely pathogenic variants within the *TSC2* gene (*p* = 0.0337). Pathogenic or likely pathogenic variants did not appear to cluster on any particular exon in either *TSC1* or *TSC2* (Figure 1). Among the eight families identified with confirmed inherited TSC, only one family (1/8) exhibited a pedigree with multiple (> 2) affected members carrying a *TSC1* pathogenic variant (Figure 2). Finally, 35% (12/34) of probands in this cohort did not have an identified pathogenic variant.

### 3.3. Characterization of the Additional Variants Identified

The initial screening identified 63 additional variants, which were categorized into two distinct groups. The pathogenic variant identified (PVI) group (19 probands) comprised 35 variants that co‐occurred with a TSC‐causing or likely causing variant (Table S4), whereas the NPVI group (12 probands) consisted of 28 variants found in patients lacking a primary molecular diagnosis (Table S5). Of the 63 total variants detected (17 in *TSC1* and 46 in *TSC2*), four variants from the PVI group were novel and had not been previously documented in public genomic databases, whereas 17 were common to both groups. Comprehensive screening of all variants against the LOVD and ClinVar databases revealed that a significant proportion is characterized as benign/likely benign variants (Tables S4 and S5).

### 3.4. Pathogenicity Assessment of VUS

To evaluate the clinical relevance of VUS, computational analysis was performed using the CanVar‐UK v2.2 and VarSome search engines. The variants suggested as likely pathogenic were further characterized using additional computational programs.

#### 3.4.1. The NM_000368.4 (*TSC1*): c.2302C>T, p.(Arg768Cys) Variant

This missense variant, identified in Patient 7 who fulfilled the definite clinical diagnostic criteria, was classified as likely pathogenic according to ACMG/AMP guidelines [10]. This classification is supported by four criteria: PM2, as the variant is extremely rare in gnomAD (GAF 2.48 × 10^−6^, Grpmax FAF 6.8 × 10^−7^); PM1, as the substitution occurs within the critical coiled‐coil domain essential for the formation of the TSC1–TSC2–TBC1D7 complex [14]; PP1, based on segregation with a mild phenotype in the parent; and PP3, in which in silico predictions were as follows: PolyPhen‐2 was 0.987 classifying the variant as probably damaging, whereas the SIFT prediction was 0.01 (with scores ≤ 0.05 being considered deleterious), indicating that the substitution of Arg to Cys affects protein function based on sequence homology and the physical properties of amino acids. Further computational characterization using the I‐TASSER server predicted a secondary structure of alpha‐helix for this region. The predicted normalized B‐factor was −0.39, a value indicative of high structural rigidity and stability, typically found in the protein′s core or alpha helices and often forms the structural scaffolding [15]. Moreover, predictive modeling assigned a solvent accessibility score of 1 to Residue 768, identifying it as a buried residue within the protein′s hydrophobic core. Given the buried nature and low B‐factor of this site, such a loss of side‐chain volume within the hydrophobic core likely compromises the structural integrity and stability of the TSC1 homodimer.

#### 3.4.2. The NM_000548.3 (*TSC2*): c.3083_3091del, p.(Asp1028_Met1030del) Variant

This in‐frame deletion of three amino acids, identified in Patient 24 who fulfilled the definite clinical diagnostic criteria, was classified as likely pathogenic according to ACMG/AMP guidelines [10]. Supporting evidence includes PM2, its absence from large population databases; PM4, the occurrence of an in‐frame deletion in a nonrepetitive region; PP3, high evolutionary conservation (phyloP100 = 9.92); and PP1, cosegregation with the disease phenotype.

In addition to the above, the following four variants were classified as VUS according to ACMG/AMP guidelines (PM2, PP3, and PP1):

NM_000548.5 (*TSC2*): c.5383C>T, p.(Arg1795Cys): identified in Probands 2 and 3.

NM_000548.5 (*TSC2*): c.3395C>T, p.(Ser1132Leu): identified in Proband 35.

NM_000548.5 (*TSC2*): c.1819G>A, p.(Ala607Thr): identified in Proband 31.

NM_000548.5 (*TSC2*): c.2664G>C, p.(Leu888=): identified in Proband 5.

### 3.5. Genotype–Phenotype Correlations

The cohort comprised 53% males and 47% females; however, no statistically significant difference in the distribution of clinical features was observed between sexes. The most prevalent manifestations (prevalence > 50%) were hypomelanotic macules (HM) and cortical dysplasias (CD) (62% each), followed by subependymal nodules (SENs) and infantile spasms (IS) (56% each).

#### 3.5.1. PVI Versus NPVI Group

Comparison of clinical features between patients with a PVI (PVI group) and those with NPVI (NPVI group) presented some trends (Table S6). The PVI group exhibited a significantly higher frequency of HM (77% vs. 31%; *p* = 0.0248) compared with the NPVI group. Although other clinical features, including SENs, CD, and IS, showed higher frequencies in the PVI group, these differences did not reach statistical significance. Conversely, lymphangioleiomyomatosis (LAM) was not observed in the PVI group (0% [0/22]) but was present in 33% (4/12) of the NPVI group (*p* = 0.0107).

#### 3.5.2. *TSC1* Versus *TSC2* Disease

Consistent with previous reports [11, 16, 17], our analysis indicated that *TSC2*‐associated disease exhibited a more severe phenotype compared with *TSC1*‐associated disease. Specifically, the TSC2 group showed a higher prevalence of renal angiomyolipomas (AML) compared with the *TSC1* group (60% vs. 0%; *p* = 0.0167). Furthermore, higher frequencies were noted for SEN (80% vs. 43%), IS (80% vs. 29%), and subependymal giant cell astrocytomas (SEGA) (20% vs. 0%), which were associated with more pronounced TSC‐associated neuropsychiatric disorders (TAND) in the *TSC2* group, although these differences did not reach statistical significance. Notably, the prevalence of CD was similar across both groups (71% in *TSC1* vs. 67% in *TSC2*), suggesting that certain structural brain abnormalities may manifest with similar frequency regardless of whether the *TSC1* or *TSC2* locus is affected. These findings are broadly consistent with previous observations in Greek patients [11, 18], further corroborating the characteristic phenotypic tendencies associated with each genotype.

#### 3.5.3. Definite Versus Possible TSC Diagnosis

Patients meeting the criteria for a definite TSC diagnosis exhibited a more severe clinical phenotype compared with those with a possible diagnosis. Specifically, the definite diagnosis cohort demonstrated a significantly higher prevalence of CD (77% vs. 12%; *p* = 0.0021), SEN (69% vs. 12%; *p* = 0.0113), HM (73% vs. 25%; *p* = 0.0331), and facial angiofibromas (AF) (50% vs. 0%; *p* = 0.0133). Furthermore, although AML were more frequent in the definite group, this trend did not reach statistical significance (*p* > 0.05). Notably, the prevalence of IS remained comparable between the two groups (58% and 62%, respectively).

#### 3.5.4. Familial Versus De Novo TSC Cases

Clinical characteristics that tended to be more frequent in the familial cases than in the de novo subgroup included seizures (50% vs. 29%) and AF (62% vs. 36%). In contrast, the de novo cases showed a higher frequency of CD and SEN (79% vs. 50%), as well as IS (79% vs. 37%); however, none of these differences between the groups were statistically significant. Additionally, the prevalence of HM was comparable between the two subgroups (87% and 71%, respectively).

### 3.6. Probands With NPVI

In the present cohort of 34 probands, no pathogenic variants were identified in 12 individuals (35%) (NPVI subgroup). The failure to obtain a clear molecular diagnosis warrants a discussion of potential biological and clinical factors while posing a challenge for future research on rare genomic events.

#### 3.6.1. NPVI Patients With Definite TSC

Among patients meeting the definite clinical criteria for TSC, six remained NPVI (50%, 6/12), representing the true NPVI‐TSC population [19]. Five of these cases were sporadic (Probands 1, 5, 22, 23, and 30), and one was familial (Proband 27). Among these, Probands 5 and 22, in addition to their LAM diagnosis, exhibited classic TSC features, such as CD, SEN, AML, and SEGA.

#### 3.6.2. NPVI Patients With Possible TSC

Six out of eight possible TSC patients were NPVI. Diagnostic ambiguity was significantly more pronounced in the possible TSC group. In four cases (Probands 10, 14, 25, and 31), the absence of a family history combined with an attenuated phenotype suggests that these individuals may represent clinical phenocopies rather than true TSC. Specifically, two of the above four cases (Probands 14 and 25) presented with LAM. These instances are likely consistent with sporadic lymphangioleiomyomatosis (S‐LAM), where pathogenic variants are restricted to the affected lung tissue and remain undetectable in genomic DNA derived from peripheral blood [20]. The remaining two possible cases (Probands 9 and 29) reported affected relatives but displayed an attenuated phenotype.

#### 3.6.3. Technical and Biological Considerations for NPVI Status

The 35% (12/34) NPVI rate in this cohort may be attributed to three primary factors. First, the presence of clinical phenocopies—A subset of the possible TSC cases was characterized by an absence of family history and attenuated phenotypes that only marginally met clinical criteria. These individuals may represent phenotypic mimics rather than true TSC patients [21]. In such instances, the clinical presentation may stem from alternative genetic etiologies or sporadic occurrences of isolated clinical features. Second, limited genomic coverage, which restricts analysis to exonic regions and 20‐bp flanking intronic sequences. Consequently, pathogenic variants within promoters, untranslated regions (UTRs), or deep intronic regions could remain undetected. Third, low‐level somatic mosaicism, which has been shown to account for a substantial proportion of molecularly unresolved TSC cases in previous studies, remains a plausible explanation for both definite and possible TSC cases also in this study [19, 20]. When the mutant allele fraction falls below the analytical threshold of standard NGS panels (typically < 10%) [22], these low‐frequency variants remain undetectable. To achieve higher sensitivity in analysis, Tyburczy et al. [19] employed higher depth sequencing across the gene coding regions (> 500×) and expanded the reading of flanking intronic areas to a minimum of 50 nucleotides. As a result, they were able to attribute 58% (26/45) of NPVI cases to somatic mosaicism and 40% (18/45) to intronic mutations. Whenever possible, molecular analysis of pathological tissues would likely maximize diagnostic sensitivity.

## 4. Study Limitations

A number of limitations of this study should be considered. First, the targeted NGS assay has a validated analytical LoD of approximately 10% VAF; it was optimized for constitutional germline testing rather than being dedicated to mosaic variant detection; consequently, very low‐level postzygotic mosaic variants (< 10% VAF) cannot be excluded and may account for a proportion of the NPVI cases. Second, the relatively small cohort size, particularly within the genotype‐defined subgroups, limits the statistical power of genotype–phenotype analyses and may reduce the ability to detect associations involving less frequent clinical manifestations. Consequently, the observed genotype–phenotype correlations should be interpreted cautiously and considered exploratory requiring validation in larger cohorts. Third, in several subgroup analyses, multiple clinical features were compared, thus increasing the possibility of type I statistical error. Fourth, copy‐number variant (CNV) analyses and multiplex ligation‐dependent probe amplification (MLPA) tests were not systematically performed in this cohort. Therefore, large genomic rearrangements (including single‐exon deletions and duplications) involving *TSC1* or *TSC2* could not be assessed by the testing strategy applied in the present study. Finally, although computational prediction tools provided additional evidence regarding the potential impact of selected variants, functional validation studies were not performed and are required to conclusively establish their biological significance.

## 5. Conclusions

Genetic analysis of *TSC1* and *TSC2* genes in this cohort of TSC patients from Greece demonstrates a predominance of *TSC2* pathogenic variants (68%) and a significantly higher diagnostic yield in patients meeting definite clinical criteria (77%). The identification of seven novel pathogenic or likely pathogenic variants expands our knowledge of the allelic heterogeneity of TSC and contributes additional information to existing variant databases. Our findings confirm that de novo pathogenic variants predominantly affect the *TSC2* gene, whereas familial cases show an equal distribution between *TSC1* and *TSC2* variants. The relatively high proportion of patients without an identified pathogenic variant underscores the importance of considering mosaicism and noncoding pathogenic alterations in unresolved cases. Ultimately, the molecular characterization of TSC probands from Greece reinforces localized genotype–phenotype correlations while providing an essential framework for early clinical intervention, personalized genetic counseling, and the potential implementation of targeted mTOR inhibitor therapies.

## Funding

This study was supported by Novartis Hellas.

## Conflicts of Interest

The authors declare no conflicts of interest.

## Supporting Information

Additional supporting information can be found online in the Supporting Information section.

## Supporting information


**Supporting Information 1** Tables S1: *TSC1* primers.

**Supporting Information 2** Table S2: *TSC2* primers.

**Supporting Information 3** Table S3: Clinical characteristics of TSC probands from Greece in the present study.

**Supporting Information 4** Tables S4: *TSC1* and *TSC2* additional variants in TSC probands from Greece with a pathogenic variant identified (PVI).

**Supporting Information 5** Table S5: *TSC1* and *TSC2* additional variants in TSC probands from Greece with no pathogenic variant identified (NPVI).

**Supporting Information 6** Table S6: Statistical comparisons of clinical features across different proband groups.

## Data Availability

Data are available from the corresponding author upon request.
