# Supplementary material for: Molecular Characterization of TSC1 and TSC2 Variants in a Greek Cohort of Tuberous Sclerosis Complex Patients
Source: Hum Mutat. 2026 Aug 10;2026:4508699. doi: 10.1155/humu/4508699 (PMC13454889; doi:10.1155/humu/4508699)
Supplement: Supplementary file 1 [file HUMU-2026-4508699-s001.docx]

Table S4. *TSC1* and *TSC2* additional variants in TSC probands from Greece with a Pathogenic Variant Identified (PVI)

| Probands | Gene | Exon | Variant | Codon change | Variation Type | Population frequency | LOVD | | | | ClinVar | | Our data |
| --- | --- | --- | --- | --- | --- | --- | --- | --- | --- | --- | --- | --- | --- |
|  |  |  |  |  |  |  | I | II | III | IV | I | II |  |
| 4,7,8,11,12,15,16,17, 19,24,26,28,32,33 | *TSC1* | 20i | c.2626-5_2626-4dupTT | p.? | Intronic | 0.525 | 9 | + | nk | b | 11 | b | 13 |
| 4 | *TSC1* | 23 | c.3324C>T | p.(Gly1108=) | Silent | 0.166 | 18 | + | nk | b | 24 | b/lb | 1 |
| 7 | *TSC1* | 6i | c.599+51C>T | p.(=) | Intronic | not found | 2 | + | nk | b | - | - | 1 |
| 8,11,19 | *TSC1* | 22 | c.2829C>T | p.(Ala943=) | Silent | 0.0813 | 43 | + | + | b | 24 | b/lb | 3 |
| 12 | *TSC1* | 4i | c.211-29G>T | p.(=) | Intronic | not found | - | - | - | - | - | - | 1 |
| 13 | *TSC1* | 19i | c.2502+51A>G | p.(=) | Intronic | 0.0582 | 6 | + | + | b | 3 | b | 1 |
| 16 | *TSC1* | 20i | c.2626-4dupT | p.? | Intronic | 0.116 | 7 | nk | + | b | 13 | b/lb | 1 |
| 26 | *TSC1* | 10 | c.1002G>A | p.(S334=) | Silent | 0.0000479 | 5 | nk | nk | lb | 13 | b/lb | 1 |
| 26 | *TSC1* | 7 | c.532G>A | p.(Val178Ile) | Missense | 0.000168 | 5 | nk | nk | b/lb | - | - | 1 |
| 35 | *TSC1* | 22 | c.2853G>T | p.Gln951His | Missense | not found | - | - | - | - | - | - | 1 |

| Probands | Gene | Exon | Variant | Codon change | Variation Type | Population frequency | LOVD | | | | ClinVar | | Our data |
| --- | --- | --- | --- | --- | --- | --- | --- | --- | --- | --- | --- | --- | --- |
|  |  |  |  |  |  |  | I | II | III | IV | I | II |  |
| 2,3 | *TSC2* | 42 | c.5383C>T | p.Arg1795Cys | Missense | 0.00187 | 17 | + | + | b/lb | 28 | b/lb | 2 |
| 2,3 | *TSC2* | 40i | c.5161-28_5161-25delTGAG | - | Intronic | 0.0103 | 17 | + | nk | b | - | - | 2 |
| 3 | *TSC2* | 32 | c.3871G>A | p.Val1291lle | Missense | 0.000026 | 3 | + | nk | b/lb | 6 | b/lb | 1 |
| 7 | *TSC2* | 17i | c.1839+38C>A | p.(=) | Intronic | 0.00000342 | - | - | - | - | - | - | 1 |
| 7 | *TSC2* | 11 | c.1110G>A | p.(Gln370=) | Silent | 0.0108 | 18 | nk | + | b | 22 | b | 1 |
| 4,8 | *TSC2* | 39i | c.5068+43G>A | p.(=) | Intronic | 0.0000979 | 2 | nk | nk | b | - | - | 2 |
| 8,12,19 | *TSC2* | 5i | c.482-3C>T | p.? | Intronic | 0.0922 | 25 | + | + | b | 20 | b | 3 |
| 11,20,28,32 | *TSC2* | 15i | c.1600-14C>T | p.(=) | Intronic | 0.0635 | 19 | + | nk | b | 15 | b | 4 |
| 11,20,28,32 | *TSC2* | 15 | c.1578C>T | p.(Ser526=) | Silent | 0.0638 | 26 | + | + | b | 21 | b | 4 |
| 7,11,13,28 | *TSC2* | 42 | c.5397G>C | p.(Ser1799=) | Silent | 0.0884 | 21 | + | + | b | 16 | b | 3 |
| 7,11,13,20 | *TSC2* | 23 | c.2580T>C | p.(Phe860=) | Silent | 0.0786 | 21 | + | + | b | 20 | b | 3 |
| 7,11,13 | *TSC2* | 22i | c.2546-12C>T | p.(=) | Intronic | 0.0801 | 15 | + | + | b | 15 | b | 2 |
| 7,11,13,28 | *TSC2* | 23i | c.2639+44C>G | p.(=) | Intronic | 0.0935 | 13 | + | + | b | 7 | b | 3 |
| 12 | *TSC2* | 8i | c.774+49G>A | p.(=) | Intronic | not found | - | - | - | - | - | - | 1 |
| 13 | *TSC2* | 16 | c.1689C>T | p.(Ala563=) | Silent | 0.0000363 | - | - | - | - | 8 | b/lb | 1 |

| Probands | Gene | Exon | Variant | Codon change | Variation Type | Population  frequency | LOVD | | | | ClinVar | | Our data |
| --- | --- | --- | --- | --- | --- | --- | --- | --- | --- | --- | --- | --- | --- |
|  |  |  |  |  |  |  | I | II | III | IV | I | II |  |
| 16 | TSC2 | 20i | c.2220+32delG | p.(=) | Intronic | 0.00344 |  | 8 | + | nk | b | 1 | nk |
| 16 | *TSC2* | 11 | c.1100G>A | p.Arg367Gln | Missense | 0.0165 | 33 | + | + | b | 29 | b | 1 |
| 16,32 | *TSC2* | 22i | c.2546-31G>A | p.(=) | Intronic | 0.0115 | 5 | nk | + | b | 7 | b/lb | 2 |
| 17 | *TSC2* | 26 | c.2948C>G | p.Ser983Cys | Missense | 0.00000685 | - | - | - | - | 5 | b/lb | 1 |
| 19 | *TSC2* | 14 | c.1378G>A | p.Ala460Thr | Missense | 0.000721 | 18 | + | nk | b/lb | 25 | b/lb | 1 |
| 19 | *TSC2* | 15i | c.1600-39C>T | p.(=) | Intronic | 0.0886 | 10 | nk | + | b | 4 | b | 1 |
| 19 | *TSC2* | 41i | c.5260-49C>T | p.(=) | Intronic | 0.102 | 14 | + | + | b | 10 | b | 1 |
| 19 | *TSC2* | 41i | c.5260-25C>G | p.(=) | Intronic | 0.107 | 11 | + | + | b | 10 | b | 1 |
| 20 | *TSC2* | 24i | c.2743-40A>G | p.(=) | Intronic | 0.00914 | 3 | nk | + | b | 4 | b/lb | 1 |
| 35 | *TSC2* | 29 | c.3395 C>T | p.Ser1132Leu | Missense | 0.0000146 | 1 | nk | nk | nk | 3 | b/lb | 1 |

Abbreviations: p.(=): protein has not been analyzed, but no change is expected on protein level; p.? : protein has not been analyzed, an effect is expected but difficult to predict; Population frequency: Data were obtained from gnomAD v4.1, the higher frequency between the exome and genome datasets is reported; LOVD (Leiden Open Variation Database); Ι: Number of patients with the specific variant identified; - : not found; II: Found (+) in patients with a pathogenic variant identified; nk: not known; III: Found (+) in non-affected individuals; IV: Clinical significance suggested; b: benign; lb: likely benign; ClinVar (public archive of interpretations of Clinically Relevant Variants); Ι: Number of independent submissions in the database; ΙΙ: Clinical significance suggested; Our data: Number of times present in our database.
