# Supplementary material for: Molecular Characterization of TSC1 and TSC2 Variants in a Greek Cohort of Tuberous Sclerosis Complex Patients
Source: Hum Mutat. 2026 Aug 10;2026:4508699. doi: 10.1155/humu/4508699 (PMC13454889; doi:10.1155/humu/4508699)
Supplement: Supplementary file 2 [file HUMU-2026-4508699-s002.docx]

Table S5. *TSC1* and *TSC2* additional variants in TSC probands from Greece with No Pathogenic Variant Identified (NPVI)

| Probands | Gene | Exon | Variant | Codon change | Variation Type | Population  frequency | LOVD | | | | ClinVar | | Our data |
| --- | --- | --- | --- | --- | --- | --- | --- | --- | --- | --- | --- | --- | --- |
|  |  |  |  |  |  |  | I | II | III | IV | I | II |  |
| 1,5,10,14,22,25, 27,29,30 | *TSC1* | 20i | c.2626-5_2626-4dupTT | p.? | Intronic | 0.525 | 9 | + | nk | b | 11 | b | 10 |
| 1,5,30 | *TSC1* | 20i | c.2626-4dupT | p.? | Intronic | 0.116 | 7 | nk | + | b | 13 | b/lb | 3 |
| 22 | *TSC1* | 22 | c.2829C>T | p.(Ala943=) | Silent | 0.081 | 43 | + | + | b | 24 | b/lb | 1 |
| 23 | *TSC1* | 20i | c.2626-4delT | p.? | Intronic | 0.107 | 8 | nk | + | b | 1 | b | 1 |
| 23 | *TSC1* | 12i | c.1263+37C>T | p.(=) | Intronic | not found | - | - | - | - | 1 | lb | 1 |
| 25 | *TSC1* | 20i | c.2626-3C>T | p.? | Intronic | 0.0173 | 6 | + | nk | b/lb | 10 | us/b | 1 |
| 29 | *TSC1* | 19i | c.2502+51A>G | p.(=) | Intronic | 0.0582 | 6 | + | + | b | 3 | b | 1 |

| Probands | Gene | Exon | Variant | Codon change | Variation Type | Population  frequency | LOVD | | | | ClinVar | | Our data |
| --- | --- | --- | --- | --- | --- | --- | --- | --- | --- | --- | --- | --- | --- |
|  |  |  |  |  |  |  | I | II | III | IV | I | II |  |
| 1,10 | *TSC2* | 11 | c.1100G>A | p.(Gln367=) | Silent | 0.0165 | 33 | + | + | b | 29 | b | 2 |
| 5 | *TSC2* | 24 | c.2664G>C | p.(Leu888=) | Silent | 0.00000205 | - | - | - | - | 2 | us/lb | 1 |
| 5 | *TSC2* | 27i | c.3131+34C>T | p.(=) | Intronic | 0.000977 | 5 | nk | nk | b/lb | 3 | lb | 1 |
| 5,14 | *TSC2* | 4 | c.255C>T | p.(Val85=) | Silent | 0.00129 | 12 | + | + | b | 24 | b/lb | 2 |
| 5,9,30 | *TSC2* | 15i | c.1600-14C>T | p.(=) | Intronic | 0.0635 | 19 | + | + | b | 15 | b | 3 |
| 5,9,30 | *TSC2* | 15 | c.1578C>T | p.(Ser526=) | Silent | 0.0638 | 26 | + | + | b | 21 | b | 3 |
| 5,30 | *TSC2* | 42 | c.5397G>C | p.(Ser1799=) | Silent | 0.0884 | 21 | + | + | b | 16 | b | 3 |
| 5,30 | *TSC2* | 23 | c.2580T>C | p.(Phe860=) | Silent | 0.0786 | 21 | + | + | b | 20 | b | 3 |
| 5,30 | *TSC2* | 22i | c.2546-12C>T | p.(=) | Intronic | 0.0801 | 15 | + | + | b | 15 | b | 3 |
| 5,30 | *TSC2* | 23i | c.2639+44C>G | p.(=) | Intronic | 0.0935 | 13 | + | + | b | 7 | b | 3 |
| 9 | *TSC2* | 24i | c.2743-40A>G | p.(=) | Intronic | 0.00914 | 3 | nk | + | b | 4 | b/lb | 1 |
| 14 | *TSC2* | 40 | c.5106C>A | p.(Ile1702=) | Silent | 0.000893 | 10 | nk | nk | b | 21 | b/lb | 1 |

| Probands | Gene | Exon | Variant | Codon change | Variation Type | Population  frequency | LOVD | | | | ClinVar | | Our data |
| --- | --- | --- | --- | --- | --- | --- | --- | --- | --- | --- | --- | --- | --- |
|  |  |  |  |  |  |  | I | II | III | IV | I | II |  |
| 25,27,30 | *TSC2* | 5i | c.482-3C>T | p.? | Intronic | 0.0922 | 25 | + | + | b | 20 | b | 3 |
| 25,30 | *TSC2* | 15i | c.1600-39C>T | p.(=) | Intronic | 0.0886 | 10 | nk | + | b | 4 | b | 2 |
| 25,27,30 | *TSC2* | 40i | c.5161-10A>C | p.(=) | Intronic | 0.209 | 22 | + | + | b | 20 | b/lb | 3 |
| 25,27,30 | *TSC2* | 41i | c.5260-49C>T | p.(=) | Intronic | 0.102 | 14 | + | + | b | 10 | b | 3 |
| 25,27,30 | *TSC2* | 41i | c.5260-25C>G | p.(=) | Intronic | 0.107 | 11 | + | + | b | 10 | b | 3 |
| 25 | *TSC2* | 39i | c.5068+43G>A | p.(=) | Intronic | 0.0000979 | 2 | nk | nk | b | - | - | 1 |
| 27,30 | *TSC2* | 41 | c.5202T>C | p.(Asp1734=) | Silent | 0.179 | 29 | + | + | b | 20 | b | 2 |
| 31 | *TSC2* | 9i | c.849-24C>T | p.(=) | Intronic | 0.000403 | 5 | + | nk | b | 1 | nk | 1 |
| 31 | *TSC2* | 17 | c.1819G>A | p.Ala607Thr | Missense | 0.000902 | 14 | nk | + | b/lb | 18 | b/lb | 1 |

Abbreviations: p.(=): protein has not been analyzed, but no change is expected on protein level; p.? : protein has not been analyzed, an effect is expected but difficult to predict; Population frequency: Data were obtained from gnomAD v4.1, the higher frequency between the exome and genome datasets is reported; LOVD (Leiden Open Variation Database); Ι: Number of patients with the specific variant identified; - : not found; II: Found (+) in patients with a pathogenic variant identified; nk: not known; III: Found (+) in non-affected individuals; IV: Clinical significance suggested; b: benign; lb: likely benign; ClinVar (public archive of interpretations of Clinically Relevant Variants); Ι: Number of independent submissions in the database; ΙΙ: Clinical significance suggested; us: uncertain significance; Our data: Number of times present in our database.
