# Supplementary material for: Molecular Characterization of TSC1 and TSC2 Variants in a Greek Cohort of Tuberous Sclerosis Complex Patients
Source: Hum Mutat. 2026 Aug 10;2026:4508699. doi: 10.1155/humu/4508699 (PMC13454889; doi:10.1155/humu/4508699)
Supplement: Supplementary file 3 [file HUMU-2026-4508699-s003.docx]

Table S3. Clinical characteristics of TSC probands from Greece in the present study

|  |  |  |  | **Neurological findings** | | | | **Renal**  **findings** | | **LAM** | **Cardiovascular**  **findings** | **Ophthalmological**  **findings** | | **Dermatological**  **findings** | | | | | | **Oral findings** | **TSC-Associated Neuropsychiatric Disorders** | | | |
| --- | --- | --- | --- | --- | --- | --- | --- | --- | --- | --- | --- | --- | --- | --- | --- | --- | --- | --- | --- | --- | --- | --- | --- | --- |
| P/  ID | Sex/Age* | Gene | TSC - DS | CD | SEGA | SEN | IS/S | AML | MRC |  | CR | MRH | RAP | AF | SP | HM | CM | CSL | UF | IF | LD | MR | ASD | ADHD |
| 1 | F/1 | - | Definite | + |  | + | IS |  |  |  |  | + |  |  |  | + |  |  |  |  |  |  |  |  |
| 2 | F/2 | *TSC1* | Definite | + |  | + |  |  |  |  |  |  |  |  |  | + |  |  |  |  |  |  |  |  |
| 3 | M/22 | *TSC2* | Definite |  |  |  |  | + |  |  |  |  |  | + |  | + |  |  |  |  |  |  |  |  |
| 4 | M/44 | *TSC2* | Definite |  |  |  | IS | + |  |  |  |  |  | + |  | + |  |  | + |  |  |  |  |  |
| 5 | F/38 | *-* | Definite | + |  | + |  | + |  | + |  |  |  |  |  |  |  |  |  |  |  |  |  |  |
| 7 | M/10 | *TSC1* | Definite | + |  |  |  |  | + |  |  |  |  | + |  |  |  |  |  |  |  |  |  |  |
| 8 | F/35 | *TSC1* | Definite | + |  |  | IS/+ |  | + |  |  |  |  | + |  |  |  |  |  |  |  |  |  |  |
| 9 | M/1 | *-* | Possible | + |  |  | IS |  |  |  |  |  |  |  |  |  |  |  |  |  |  |  |  |  |
| 10 | M/10 | *-* | Possible |  | + |  | IS |  |  |  |  |  |  |  |  |  |  |  |  |  |  |  |  |  |
| 11 | F/35 | *TSC1* | Definite | + |  |  | + |  |  |  |  |  |  | + |  | + |  |  | + |  | + |  |  |  |
| 12 | M/15 | *TSC2* | Definite |  | + | + | IS |  |  |  |  | + |  |  |  | + |  |  |  |  |  |  |  |  |
| 13 | M/13 | *TSC2* | Definite | + |  | + | IS | + | + |  |  | + |  |  |  | + |  |  |  |  | + |  |  |  |
| 14 | F/38 | *-* | Possible |  |  |  | + |  |  | + |  |  |  |  |  |  |  |  |  |  |  |  |  |  |
| 15 | M/6 | *TSC1* | Definite | + |  | + |  |  |  |  |  |  |  |  |  | + |  |  |  |  |  |  |  |  |
| 16 | M/30 | *TSC2* | Definite | + |  | + | IS/+ | + |  |  |  |  | + | + |  | + |  |  | + |  | + | + | + |  |
| 17 | F/11 | *TSC2* | Definite | + |  | + | IS/+ | + |  |  |  | + |  | + |  |  |  |  |  |  |  | + |  | + |
| 18 | M/10 | *TSC2* | Definite | + | + | + | IS/+ | + | + |  | + | + |  | + |  | + |  |  |  | + |  | + |  | + |
| 19 | M/5m | *TSC2* | Definite | + |  | + | IS |  |  |  | + |  |  |  |  | + |  |  |  |  |  |  |  |  |
| 20 | F/1 | *TSC2* | Possible |  |  | + | IS |  | + |  |  |  |  |  |  |  |  |  |  |  |  |  |  |  |
| 21 | F/6 | *TSC2* | Definite | + |  | + | IS |  |  |  | + | + |  |  |  | + |  |  |  |  |  |  |  |  |
| 22 | M/8 | *-* | Definite | + | + | + | IS/+ | + |  | + |  |  |  |  |  | + |  |  |  |  | + |  |  | + |
| 23 | M/22 | *-* | Definite | + |  | + |  |  |  |  |  |  |  | + |  | + |  |  | + |  |  |  |  |  |
| 24 | M/11 | *TSC2* | Definite | + |  | + | + | + |  |  | + |  |  | + | + | + |  | + | + |  | + | + | + |  |
| 25 | F/43 | - | Possible |  |  |  |  | + |  | + |  |  |  |  |  |  |  |  |  |  |  |  |  |  |
| 26 | F/20 | *TSC2* | Definite | + |  | + | IS/+ | + |  |  |  |  |  | + |  | + | + |  |  |  | + |  |  |  |
| 27 | M/40 | *-* | Definite | + |  |  |  | + |  |  |  |  |  | + |  |  |  |  |  |  |  |  |  |  |
| 28 | M/8m | *TSC1* | Possible |  |  |  | IS |  |  |  |  |  |  |  |  | + | + |  |  |  |  |  |  |  |
| 29 | M/3 | *-* | Possible |  |  |  | IS |  |  |  |  |  |  |  |  |  |  |  |  |  |  |  |  |  |
| 30 | F/40 | *-* | Definite |  |  |  |  | + |  |  |  |  |  | + |  |  |  |  |  |  |  |  |  |  |
| 31 | F/20 | *-* | Possible |  |  |  | + |  |  |  |  |  |  |  |  | + |  |  |  |  |  |  |  |  |
| 32 | F/9 | *TSC2* | Definite | + | + | + | IS | + |  |  |  |  |  |  |  | + | + |  |  |  | + |  |  |  |
| 33 | F/5 | *TSC1* | Definite |  |  | + | + |  |  |  |  |  |  |  |  | + |  |  |  |  |  |  |  |  |
| 34 | M/1 | *TSC2* | Definite | + findings |  |  | IS |  |  |  |  |  |  |  |  | + |  |  |  |  |  |  |  |  |
| 35 | F/6m | *TSC2* | Definite |  |  | + |  |  |  |  | + |  |  |  |  |  |  |  |  |  |  |  |  |  |

Abbreviations: P/ID (Proband ID): Each number corresponds to the specific proband; * Age of diagnosis in years or months; CD: Cortical dysplasias; SEGA: Subependymal giant cell astrocytomas; SEN: Subependymal nodules; S/IS: Seizures/Infantile spasms; AML: Renal angiomyolipomas; MRC: Multiple renal cysts; LAM: Lymphangioleiomyomatosis; CR: Cardiac rhabdomyomas; MRH: Multiple retinal hamartomas; RAP: Retinal achromic patch; AF: Angiofibroma; SP: Shagreen patch; HM: Hypomelanotic macules; CM: Café au lait macules; CSL: Confetti skin lesions; UF: Ungual fibromas; IF: Intraoral fibromas; LD: Learning disabilities; MR: Mental retardation; ASD: Autism spectrum disorder; ADHD: Attention Deficit Hyperactivity Disorder.
