# Supplementary material for: Molecular Characterization of TSC1 and TSC2 Variants in a Greek Cohort of Tuberous Sclerosis Complex Patients
Source: Hum Mutat. 2026 Aug 10;2026:4508699. doi: 10.1155/humu/4508699 (PMC13454889; doi:10.1155/humu/4508699)
Supplement: Supplementary file 4 [file HUMU-2026-4508699-s004.docx]

Table S1. *TSC1* Primers

| Exon | Forward 5΄- 3΄ | Reverse 5΄- 3΄ |
| --- | --- | --- |
| 3 | GCATTAGTTTGTCTTGCAGG | TATCAGCAGGATTCTAGTGG |
| 4 | CTGGATTTTGTGACAGGAAGC | GCTCAGGACAAGTTGCACAG |
| 5 | AGACAAGACTTGAGAGATTGG | AGCTTCCTTGCTTTAAGTTGC |
| 6 | TCAGTGTTTAGAGCCTCTTC | ATTCCCTCATCTGTCTGGTG |
| 7 | GGAGTGAGGTGGGAATATAC | AGTGGCGAGGAAGAAAACTG |
| 8 | GTTCTCAAGGTTCCATCTGTG | TCCCAAATATACCTCAACAGG |
| 9 | GCTTGTTCTTTGCTATCAGAG | TAGGAACTGAACTAAGTCTTAC |
| 10 | CTCTGAAGTGTTACTTTGTCC | GATCCTTAAAAGTGACTCCTG |
| 11 | CATGGATATAAACCTCGTGGA | CACTTTACACATTCTGAAAGC |
| 12 | TCAGAATGTGTAAAGTGAACC | TCAAACCCATTGCATTTTAGG |
| 13 | ATTTGAGAATCACTGCACTCG | CCCAGAATTTCCTTGTTTCCA |
| 14 | TAAGCAGTTTTACACTTACAGG | GTGAGCAATGGCACAAAATCC |
| 15A | TCTCCTCTCTCTGAGTGACA | GGGAGTAAAGGCTTGCTTTG |
| 15B | ACTCGGCAGCCTCCAGTTC | CTTTTGCTTTCTTTAACAGCTC |
| 15C | CGACGAGAGTGGGCTTTGG | CTGCCTCAAGGTGGGAGTG |
| 16 | GCCATTGCAGATTTTGACCAC | CTGTTTCCCAGAGGGCACC |
| 17 | AAGCTAACAACACATGGGAAG | CCAAAGGAGTGGGAAGGAC |
| 18 | TATTCAGATGAAATGTTCGCAG | AACAAGTCAAGGACACCCAG |
| 19 | AGAAAGTAGAGCCGTTGAGC | CCATGACACAGACACTCAAG |
| 20 | AACATTGAAATAGTGTGCTCC | CATCAAGCCATTCTCTATGC |
| 21 | ACTAAAATGCCTTCTCAGTCC | GAAAGATACAGACCAGCCAG |
| 22 | TCATCAGGAAAGGCTTTTGTG | CCTGTCTAGTCAGCAGTAAC |
| 23A | AGTGAGTGTCAGTGTAATTCC | CTTCTCCCATAGTCGTCTCC |
| 23B | AGCAGCAGCGAGCTTTCTAC | ACACCCAAGTCTTTGCCCAG |
| 23C | TTTCGTAATAAGAGCGAGAGC | TTTGAAACGTGCATTCACACC |
