# Supplementary material for: Molecular Characterization of TSC1 and TSC2 Variants in a Greek Cohort of Tuberous Sclerosis Complex Patients
Source: Hum Mutat. 2026 Aug 10;2026:4508699. doi: 10.1155/humu/4508699 (PMC13454889; doi:10.1155/humu/4508699)
Supplement: Supplementary file 5 [file HUMU-2026-4508699-s005.docx]

Table S6. Statistical comparisons of clinical features across different proband groups

|  | **Total  (N = 34)** | ***TSC1* (N = 7)** | ***TSC2*  (N = 15)** | **PVI  (N = 22)** | **NPVI  (N = 12)** | **Definite (N = 26)** | **Possible (N =8)** | **Familial (N = 8)** | ***De novo* (N = 14)** | ***TSC1***  **vs. *TSC2*** | **PVI**  **vs.**  **NPVI** | **Definite**  **vs.**  **Possible** | **Familial vs. *De novo*** |
| --- | --- | --- | --- | --- | --- | --- | --- | --- | --- | --- | --- | --- | --- |
|  |  |  |  |  |  |  |  |  |  | p < 0.05 | p < 0.05 | p < 0.05 | p < 0.05 |
| **Male** | 18 (53%) | 3 | 9 | 12 | 6 | 14 | 4 | 5 (62%) | 7 (50%) | ns | ns | ns | ns |
| **Female** | 16 (47%) | 4 | 6 | 10 | 6 | 12 | 4 | 3 (38%) | 7 (50%) | ns | ns | ns | ns |
| **CD** | 21 (62%) | 5 (71%) | 10 (67%) | 15 (68%) | 6 (50%) | 20 (77%) | 1 (12%) | 4 (50%) | 11 (79%) | ns | ns | 0.0021 | ns |
| **SEN** | 19 (56%) | 3 (43%) | 12 (80%) | 15 (68%) | 4 (33%) | 18 (69%) | 1 (12%) | 4 (50%) | 11 (79%) | ns | ns | 0.0113 | ns |
| **SEGA** | 5 (15%) | 0 (0%) | 3 (20%) | 3 (14%) | 2 (17%) | 4 (15%) | 1 (12%) | 1 (12%) | 2 (14%) | ns | ns | ns | ns |
| **Seizures** | 11 (32%) | 3 (43%) | 5 (33%) | 8 (36%) | 3 (25%) | 9 (35%) | 2 (25%) | 4 (50%) | 4 (29%) | ns | ns | ns | ns |
| **IS** | 19 (56%) | 2 (29%) | 12 (80%) | 14 (64%) | 5 (42%) | 14 (54%) | 5 (62%) | 3 (37%) | 11 (79%) | ns | ns | ns | ns |
| **LD** | 7 (21%) | 1 (14%) | 5 (33%) | 6 (27%) | 1 (8%) | 7 (27%) | 0 (0%) | 3 (37%) | 3 (21%) | ns | ns | ns | ns |
| **MR** | 4 (12%) | 0 (0%) | 4 (27%) | 4 (18%) | 0 (0%) | 4 (15%) | 0 (0%) | 1 (12%) | 3 (21%) | ns | ns | ns | ns |
| **ASD** | 2 (6%) | 0 (0%) | 2 (13%) | 2 (9%) | 0 (0%) | 2 (8%) | 0 (0%) | 1 (12%) | 1 (7%) | ns | ns | ns | ns |
| **ADHD** | 3 (9%) | 0 (0%) | 2 (13%) | 2 (9%) | 1 (8%) | 3 (11%) | 0 (0%) | 0 (0%) | 2 (14%) | ns | ns | ns | ns |
| **AML** | 14 (41%) | 0 (0%) | 9 (60%) | 9 (41%) | 5 (42%) | 13 (50%) | 1 (12%) | 3 (37%) | 6 (43%) | 0.0167 | ns | ns | ns |
| **MRC** | 5 (15%) | 2 (29%) | 3 (20%) | 5 (23%) | 0 (0%) | 4 (15%) | 1 (12%) | 1 (12%) | 4 (29%) | ns | ns | ns | ns |
| **LAM** | 4 (12%) | 0 (0%) | 0 (0%) | 0 (0%) | 4 (33%) | 2 (8%) | 2 (25%) | 0 (0%) | 0 (0%) | ns | 0.0107 | ns | ns |
| **CR** | 5 (15%) | 0 (0%) | 5 (33%) | 5 (23%) | 0 (0%) | 5 (19%) | 0 (0%) | 1 (12%) | 4 (29%) | ns | ns | ns | ns |
| **MRH** | 6 (18%) | 0 (0%) | 5 (33%) | 5 (23%) | 1 (8%) | 6 (23%) | 0 (0%) | 1 (12%) | 4 (29%) | ns | ns | ns | ns |
| **RAP** | 1 (3%) | 0 (0%) | 1 (7%) | 1 (4%) | 0 (0%) | 1 (4%) | 0 (0%) | 0 (0%) | 1 (7%) | ns | ns | ns | ns |
| **AF** | 13 (38%) | 3 (43%) | 7 (47%) | 10 (45%) | 3 (25%) | 13 (50%) | 0 (0%) | 5 (62%) | 5 (36%) | ns | ns | 0.0133 | ns |
| **SP** | 1 (3%) | 0 (0%) | 1 (7%) | 1 (4%) | 0 (0%) | 1 (4%) | 0 (0%) | 1 (12%) | 0 (0%) | ns | ns | ns | ns |
| **HM** | 21 (62%) | 5 (71%) | 12 (80%) | 17 (77%) | 4 (33%) | 19 (73%) | 2 (25%) | 7 (87%) | 10 (71%) | ns | 0.0248 | 0.0331 | ns |
| **CM** | 3 (9%) | 1 (14%) | 2 (13%) | 3 (14%) | 0 (0%) | 2 (8%) | 1 (12%) | 2 (25%) | 1 (7%) | ns | ns | ns | ns |
| **CSL** | 1 (3%) | 0 (0%) | 1 (7%) | 1 (4%) | 0 (0%) | 1 (4%) | 0 (0%) | 1 (12%) | 0 (0%) | ns | ns | ns | ns |
| **UF** | 5 (15%) | 1 (14%) | 3 (20%) | 4 (18%) | 1 (8%) | 5 (19%) | 0 (0%) | 2 (25%) | 2 (14%) | ns | ns | ns | ns |
| **IF** | 1 (3%) | 0 (0%) | 1 (7%) | 1 (4%) | 0 (0%) | 1 (4%) | 0 (0%) | 0 (0%) | 1 (7%) | ns | ns | ns | ns |

Abbreviations: PVI: Pathogenic variant identified; NPVI: No pathogenic variant identified; CD: Cortical dysplasias; SEN: Subependymal nodules; SEGA: Subependymal giant cell astrocytomas; IS/S: Infantile spasms/Seizures; LD: Learning disabilities; MR: Mental retardation; ASD: Autism spectrum disorder; ADHD: Attention Deficit Hyperactivity Disorder; AML: Renal angiomyolipomas; MRC: Multiple renal cysts; LAM: Lymphangioleiomyomatosis; CR: Cardiac rhabdomyomas; MRH: Multiple retinal hamartomas; RAP: Retinal achromic patch; AF: Angiofibroma; SP: Shagreen patch; HM: Hypomelanotic macules; CM: Café au lait macules; CSL: Confetti skin lesions; UF: Ungual fibromas; IF: Intraoral fibromas; ns: No statistically significant difference.
