# Supplementary material for: Molecular Characterization of TSC1 and TSC2 Variants in a Greek Cohort of Tuberous Sclerosis Complex Patients
Source: Hum Mutat. 2026 Aug 10;2026:4508699. doi: 10.1155/humu/4508699 (PMC13454889; doi:10.1155/humu/4508699)
Supplement: Supplementary file 6 [file HUMU-2026-4508699-s006.docx]

Table S2. *TSC2* Primers

| Exon | Forward 5΄- 3΄ | Reverse 5΄- 3΄ |
| --- | --- | --- |
| 1 | GTCCCCATTCCTGTTTCGTT | TCTCTGCATTTCCCTCTAGC |
| 2 | TGGAAAATGCAGTGGGAGTC | GCAAACCAGATCATCGGCAG |
| 3 | GTGGGGCTCTCAGTCACAAG | TAGACCCTGTGCCCAGCAAC |
| 4 | CTGCACTTCAGGGACTTCTTG | CATCCAGGTTACACTTGATCG |
| 5 | GACTGATGATGGGGTTTCTG | ACTGCGGAGCTGAACTTAGG |
| 6 | ACGTCATAGAGTGACTAGACC | CTGAGGCTCAGAGAGACCGA |
| 7 | TGAAGGAGGTGGGAAGGAAG | AGACAACCATTCATGGGAGAC |
| 8 | GTCTTTGGGAGGAGATGGTG | GTGTGTGCTTGGCAAGGGAC |
| 9 | CCTCGGCAACCTCACACATC | TTCCTGCCAGCTCACTGCAC |
| 10 | ATAAACGTGTGGTGGGCACTG | ATCTTCTGAGTGGGCCGAGC |
| 11 | GGCTGAGGGTGTCTCCATGC | TTCTGAGGCTCAGAAAGCTGC |
| 12 | CTGGGCTCTGACAGCAAACC | CTCATCGGGCATCCTGAGAC |
| 13 | CTCTGTGCCCTGTGTGCCTG | CACGGAGAAATAGCCCTGAGC |
| 14 | GGTCGGTTCCTGAGGAATTGG | GAGCTCTGGCACGCTAGCAC |
| 15 | AGGTGTTTGTGGTAGAAAGTG | GGAACAGACTCCAACACAAC |
| 16 | GTGTTTTGAAGCACGCACTCTA | GGAACCACACCTGCCGAGAC |
| 17 | GTGTCTGTGTTGGGATGTGG | CCCTCAGACATGCAAACAGC |
| 18 | GCGCTGTTTGCATGTCTGAGG | AGGCCAGACAGGGAGGGAG |
| 19 | CTGAGAACAGGGCTCCATAG | CAGAGGCTCGCAGTCTTTTG |
| 20 | CACTCCCACCACTCCGAAAG | CATCAAGCTCGCCTGTCCTG |
| 21 | TTCAGGGACTTGCTAAGCCTC | GCACAGGTTACACAGCCCGA |
| 22 | CGTGGCCTTCTCTCCTCTG | CCTTGGGGACAAAGGCTGC |
| 23 | CTTTGTCCCCAAGGCCTGAG | CAAAGCAAGCCCTGGCCTAC |
| 24 | CCTGCAGCTTGTCCCTGG | CTCACTGACTTGGAGGCAC |
| 25 | CTCTCCATCCTGACCCTGTG | CAGACGATGAGGTCATGCAAG |
| 26 | CTGTTGGGGTCTTTCCGAGC | ACAGGGAGCGTGAAACCCAG |
| 27 | CTGGGTTTCACGCTCCCTGT | TTAGTCCCCAGGCTGGTACG |
| 28 | TGACTGCACCTTCCTTTCCTC | ACACTGGGACCAGCAGCAAC |
| 29 | TCTGGCTGCCTGTGGCACTA | AAGAGGGCCAAGTCTGCAATC |
| 30 | TGAGGATTGTGGGAGGGAGC | GCAGAGAGGAGCCACATTGC |
| 31 | CAGCTTGTAGCTAGCACTGG | ACAGCACCTATGAGGCAGAG |
| 32 | TGTCCCTCTGGTCAGGAGAAG | AGAGCCCTGCCTCCCCTAAG |
| 33A | CTGGGATGGAGGACAGATAG | TGACCGGGCCTTAACCTCAG |
| 33B | CCTCTGTGGACCTCTCCTTC | TACTCTCTTGCCCCTGCGTG |
| 33C | GTTAAGGCCCGGTCACAGTC | CAAGTGAGGCACCAGGTTCC |
| 34 | CGGTGGAGTGGGAGATGG | GAGCCCTCTGCAAGCCTG |
| 35 | GGAGCTCAGGCTTGCAGAGG | ACTTCATGCTGTAGGGGACC |
| 36 | CTGCTGGAATGGATGGTCTTG | GTAGCAGGACTGGATGGGAG |
| 37 | CTGGAGTAATCAGGAGGTGC | CGAAGCTCAGGGAGGAGG |
| 38 | TGCCAGAGGGGAAAGTTCAG | GGGAGACTTGGTGGTCACG |
| 39 | TGCAGTGCAGGAAAGGTAGG | GTGAGCTCACTATCAGGTGG |
| 40 | TGGCCAAGATCGTGTCTGAC | GACAGGCAATACCGTCCAAG |
| 41 | TCACAGGTGCATCATAGCCG | GACAGGCAATACCGTCCAAG |
